# No Evidence for an Auditory Attentional Blink for Voices Regardless of Musical Expertise

**DOI:** 10.3389/fpsyg.2019.02935

**Published:** 2020-01-10

**Authors:** Merve Akça, Bruno Laeng, Rolf Inge Godøy

**Affiliations:** ^1^RITMO Center for Interdisciplinary Studies in Rhythm, Time and Motion, University of Oslo, Oslo, Norway; ^2^Department of Musicology, University of Oslo, Oslo, Norway; ^3^Department of Psychology, University of Oslo, Oslo, Norway

**Keywords:** attentional blink, temporal selective attention, perceptual expertise, musical expertise, auditory attention

## Abstract

**Background:** Attending to goal-relevant information can leave us metaphorically “blind” or “deaf” to the next relevant information while searching among distracters. This temporal cost lasting for about a half a second on the human selective attention has been long explored using the attentional blink paradigm. Although there is evidence that certain visual stimuli relating to one's area of expertise can be less susceptible to attentional blink effects, it remains unexplored whether the dynamics of temporal selective attention vary with expertise and objects types in the auditory modality.

**Methods:** Using the auditory version of the attentional blink paradigm, the present study investigates whether certain auditory objects relating to musical and perceptual expertise could have an impact on the transient costs of selective attention. In this study, expert cellists and novice participants were asked to first identify a target sound, and then to detect instrumental timbres of cello or organ, or human voice as a second target in a rapid auditory stream.

**Results:** The results showed moderate evidence against the attentional blink effect for voices independent of participants' musical expertise. Experts outperformed novices in their overall accuracy levels of target identification and detection, reflecting a clear benefit of musical expertise. Importantly, the musicianship advantage disappeared when the human voices served as the second target in the stream.

**Discussion:** The results are discussed in terms of stimulus salience, the advantage of voice processing, as well as perceptual and musical expertise in relation to attention and working memory performances.

## 1. Introduction

Attentional blink (AB; Raymond et al., 1992) refers to the phenomenon that when two targets are presented in close temporal proximity, report of the second target (T2) is often impaired after correctly identifying the first target (T1). The AB is known to be an effective tool to study the time-course of selective attention and has been studied extensively in the visual domain using a wide range of material. However, to date, relatively few studies have investigated its auditory counterpart. Those investigations have shown evidence for the auditory AB effect (but see Potter et al., 1998 for conflicting evidence) using simple, non-musical tones (e.g., Shen and Mondor, 2006; Shen and Alain, 2012), spoken letters and digits (e.g., Arnell and Larson, 2002; Arnell and Jenkins, 2004; Martens et al., 2015), and spoken syllables (e.g., Duncan et al., 1997; Tremblay et al., 2005) as auditory stimuli. The aim of the present study is to investigate temporal dynamics of selective auditory attention and whether these dynamics can be modulated by an individual's expertise and the objects of this expertise. We focused in particular on common perceptual expertise with voices and musical expertise. Here the perceptual expertise with voices refers to the common human experience that is associated with higher level of perceptual capacity for voices, shaped by extensive exposure to human voices for years on a daily basis, while the musical expertise refers to advanced levels of music performance experience combined with extensive training in music. Employing the auditory version of the attentional blink paradigm, we explored the temporal costs of selective attention among expert cellist and novice participants for different auditory objects through systematically manipulating the type of the second target (human voice, cello, organ) and the interval between the first and second target (i.e., lag) presented in a rapid auditory stream.

Only recently has it been demonstrated that AB is not as universal of a cognitive limitation as once was thought, but that it greatly differs between individuals, or groups of individuals, and these differences in the AB frequency are dependent on various factors, such as stimulus category, duration, and modality (Willems and Martens, 2016). Previous studies also illustrated that certain visual stimuli, such as faces (Awh et al., 2004; Landau and Bentin, 2008), which constitute an expertise object for almost all of us, and other expertise objects (e.g., cars for car experts, Blacker and Curby, 2016) can lower one's susceptibility to the AB effects. Less well-understood is whether this kind of expertise-related alleviation in the AB effects can be observed in the auditory domain, since the few existing studies regarding auditory AB did not explore this factor.

In this study human voices were used as it is believed that they are, as the human faces in the visual modality, objects of expertise for most of us. Ample evidence exists supporting the neural and cognitive similarities in the perception of human faces and voices, suggesting a unifying coding mechanism (for a review, see Yovel and Belin, 2013). It can be argued that due to our extensive experience with (and the evolutionary importance of) human voices, all humans with normal hearing abilities can be considered voice experts. To contrast the possible AB effects (or lack thereof) for human voices, instrumental tones were used, which were cello and organ tones. Interestingly, cello tones have the strongest acoustic similarity to human voices among instruments, as reported by listeners in Askenfelt (1991)'s study. Later neuroimaging studies also support that cello tones share some similarities with the human voice. For example (Levy et al., 2003), showed that string instruments elicited a larger positivity than brass and woodwind instruments, with cello evoking the largest positivity among the string instruments. The authors suggested that these findings may reflect the perceptual similarity between string instruments and human voices. Furthermore, a recent fMRI study also indicated a direct overlap on the brain networks during cello playing and during singing, as well as that playing cello may directly engage the vocal areas of the brain especially for the experienced cellists whose musical training started before the age of 7 (Segado et al., 2018). It is therefore noteworthy to explore the AB effects for cello tones not only as an expertise-object for the cellists but also as a possible expertise object for the people who has no musical training due to the similarities shared with the human voices. Organ tones were chosen as a control for both cello tones and human voices, as a contrasting timbre with an assumed common familiarity with organ tones for individuals from most Western cultures without a professional expertise as an organist.

Defined as the ability of accurate and rapid identification of individual sound sources within a set of homogenous stimuli (Chartrand et al., 2008), auditory recognition expertise has been studied mostly with musicians, and musicians in this sense are often considered as “auditory experts.” There exists evidence that musicians' auditory processing abilities differ from that of non-musicians at a behavioral level. For example, tasks involving pitch discrimination, processing of temporal information, processing of instrumental timbres, and discrimination of instrumental and voice timbres (Chartrand and Belin, 2006), all have been observed to be performed better among musicians than non-musicians. Furthermore, according to some neuroimaging studies, musician and non-musician brains seem to have differences in terms of volume, density, connectivity, morphology, and functional activity across various brain regions and structures (e.g., Gaser and Schlaug, 2003; Hyde et al., 2009). Although it is often difficult to disentangle the influences of external environmental factors from the innate biological factors, some longitudinal studies seem to suggest that the differences in musicians' and non-musicians' brain reflect their learning experiences (e.g., Hyde et al., 2009). However, it is important to note that the present cross-sectional study does not aim at differentiating training-related and genetic factors in becoming an expert musician, or asserts the observed musical expertise benefits to have a genetic or environmental basis.

The question of whether transfer of the skill, that is, the ability of specific experience to impact seemingly unrelated processes, is also explored in several studies (see Kraus and Chandrasekaran, 2010 for a review). In the context of musical training, both near transfer (i.e., benefits in highly similar contexts/domains despite the lack of training, such as better perception of piano tones in violinists) and far transfer (i.e., benefits to activities outside of the trained domain, such as language processing) effects has been documented (Moreno and Bidelman, 2014). So musical training may have benefits that are not simply limited within the scope of music field but often extends to influencing high-level cognitive functions, such as cognitive control, attention and working memory (WM). For example, a recent study pointed out that the association between music training and executive functioning was strongest for the executive functioning of WM in both visual and auditory modalities (Slevc et al., 2016). This, by itself, however does not ascertain far-transfer of musical training skills, as causality cannot be inferred from correlational studies.

Selective attention and working memory are known to share a strong link. Considering the AB literature, interaction of WM and the AB has been demonstrated behaviorally (e.g., Akyürek et al., 2007) and with brain imaging techniques (e.g., Johnston et al., 2012). Moreover, individuals with higher levels of WM functioning and broad attentional focus seem to perform better in the AB paradigm compared to those with lower WM and with narrow attentional focus (for a review see Willems and Martens, 2016). There are some documentations in the literature which may indicate better deployment of attention in time among musicians than non-musicians. For example, using tones (auditory AB task stimuli) and lines (visual AB task stimuli), Slawinski et al. (2002) reported attenuated AB in both visual and auditory modalities in musicians compared to non-musicians. Using letters and digits (presented in auditory and visual modalities), Martens et al. (2015) observed an attenuation and delay of the AB only in the auditory modality in musicians and suggested that music training have a modality-specific beneficial effect on selective attention. Despite these documentations of attenuated auditory AB in musicians, the question of whether this benefit on auditory attention can be altered using other kinds of auditory stimuli remains unanswered.

The four main research questions posed in the present experiment are as follows:

1. Are human voices less susceptible to the auditory AB effects than instrumental tones?
2. If so, does this effect extend to instruments sharing perceptual similarity to human voice (i.e., cello tones)?
3. Do expert musicians show an overall attenuation of the auditory AB as compared with novices due to their extensive auditory training with tones?
4. Do expert musicians show less auditory AB to the musical timbres associated with their principal instrument compared to timbres associated with the instruments that they have not been trained on?

Additionally, we explored whether musical sophistication (as measured by Gold-MSI inventory) and WM span (as measured with Letter-Number Sequencing task) are related to individual or group level differences in the auditory AB effect.

## 2. Materials and Methods

### 2.1. Participants

Thirty-eight volunteers (19 expert cellists and 19 novice participants) were recruited for the present study. Novice participants were defined as participants with no previous cello training and with very little or no musical training, while the expert cellists were defined as professional or advanced cello players with extensive musical training. Data from one novice participant were removed due to having a T1 identification accuracy rate below the chance level. Data from one cellist with a musical sophistication score and years of cello experience >2*SD* below the group mean was also not included in the analysis. The final sample consisted of 18 expert cellists (13 female, age range = 21–36 years, mean age = 28.89, *SD* = 5.06) and 18 novices (13 female, age range = 21–39 years, mean age = 28.17, *SD*= 5.86). The groups did not differ significantly in terms of their age [*t*_(34)_ = −0.396, *p* = 0.69] and no statistically significant relationship was observed between participant group and gender (*p* = 1.00, odds ratio = 0.00, CI%: −1.459 to 1.459. The average number of years of cello experience was 19.94 years (*SD* = 5.25, ranging from 9 to 28 years). The majority of the expert cellists was recruited from the Norwegian Academy of Music and the novices were recruited from the University of Oslo. The general exclusion criteria were having a history of hearing, speech or neurological disorders. All participants received a gift card worth of 200 NOK as compensation for their time.

### 2.2. Auditory Stimuli

Auditory stimuli consisted of 16 human voice excerpts, 16 cello tones, 16 organ tones, as well as 16 pure sine tones at various frequencies equally spaced on a logarithmic scale, white noise, and 48 different environmental sounds (e.g., broom, doorbell, motorbike). Instrumental sounds, voices, and sine tones were periodic and with harmonic spectra, while the environmental sounds and white noise were mostly non-periodic and did not have harmonic spectra. Pure tones and white noise were generated using Audacity 2.2.1 sound editing software (https://audacityteam.org/). Cello and organ (i.e., baroque plenum) instrument sounds were sampled from the McGill University Master Samples DVD set (Opolko and Wapnick, 2006), and the rest of the stimuli were sampled from freesounds.org. We have chosen 150 ms excerpts from the quasi-stationary portions of the sound (thus, avoided the initial attack transients). The excerpts were taken for cello, organ, and voice stimuli on D#2, E2, F2, G2, G#2, C3, C#3, D3, D#3, E3, F3, F#3, G3, G#3, A#3, and B3 pitches. Human voices included digital recordings of vowels sung by a male voice. Stimuli were normalized using the peak normalization method and matched by duration (150 ms with 2 ms linear amplitude ramps to eliminate the perceptual effects of onset and offset clicks). In the experimental paradigm, pure tones and white noise served as T1, human voice, cello, organ tones served as T2, and environmental sounds served as distracters.

### 2.3. Design and Procedure

**Figure 1:**
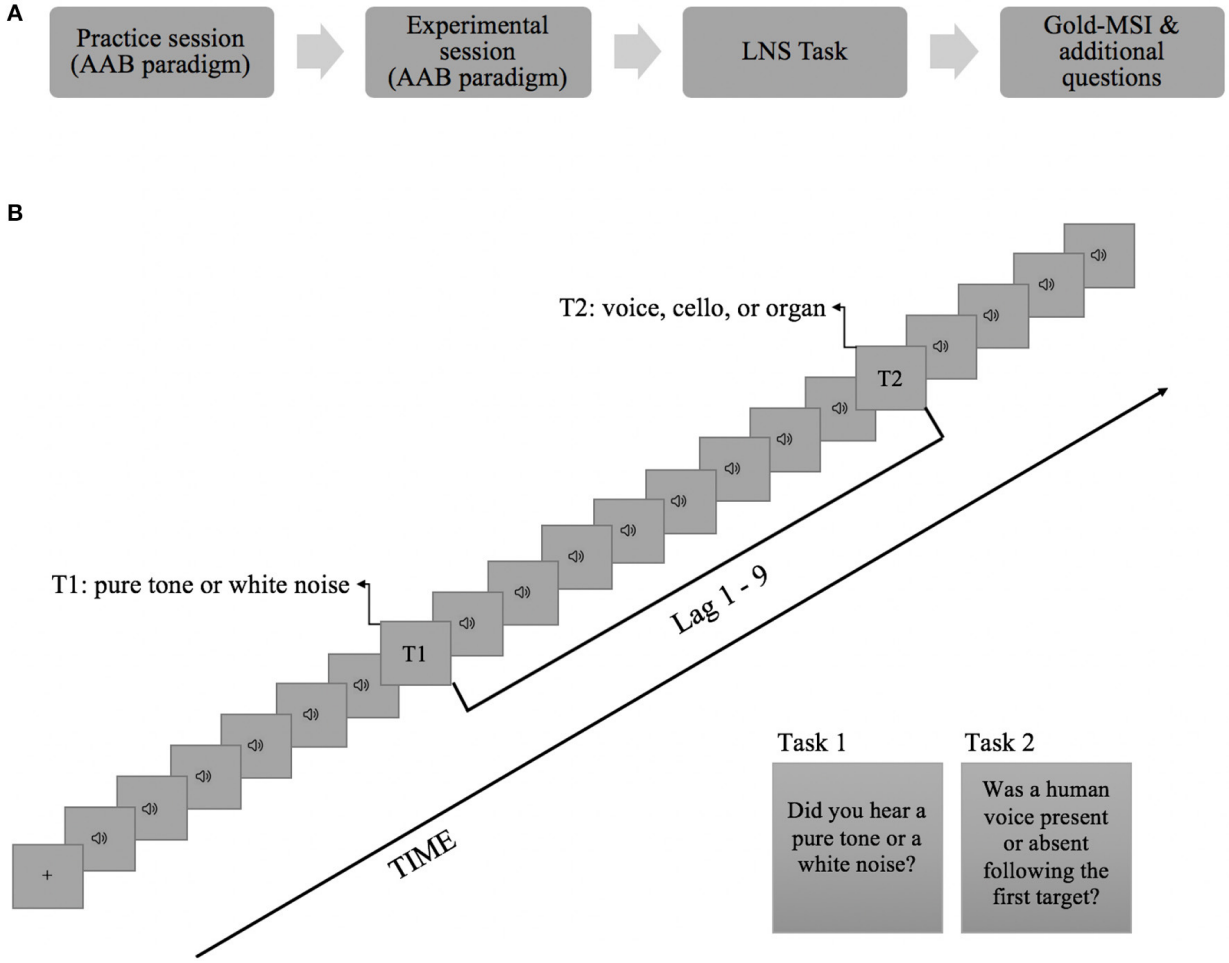
**(A)** Experimental procedure. **(B)** Schematic representation of the time-course of a trial. On every trial, 20 brief sounds were presented binaurally. Each item was presented for 150 ms followed by an interstimulus interval of 10 ms. The first target could occur either as the 5th or the 7th item in the stream and the interval between T1 and T2 (i.e., lag) was manipulated to represent 1, 2, 3, or 9 temporal positions following T1. The participants had to report T1 and T2 at the end of the trial with key presses. This example depicts a trial where T1 was presented as the 7th item in the RAP stream with a nine stimulus interval (Lag 9) between T1 and T2.

The experiment took place at the cognitive laboratory in the Department of Psychology in the University of Oslo. It started with a practice session which then was followed by an experimental session of the AB task. E-Prime 3.0 software (Psychology Software Tools, Pittsburgh, PA, USA) was used for the presentation of the stimuli and for response collection in the AB task. After completion of the AB task, the Letter-Number Sequencing task and the Musical Sophistication Index were administered (see Figure 1A for an illustration of the experimental procedure). The practice session consisted of 16 trials representing all T2 categories and the participants were given accuracy feedback at the end of every trial. In the experimental session, the participants went through a total of 192 trials with three alternating blocks of all T2 types. The order of the blocks was counterbalanced across participants. No feedback was provided to the participants during the experimental session.

Figure 1B depicts an example trial from the AB task. Each trial began with a fixation cross (+) appearing at the center of the screen for 250 ms. Then, a rapid auditory presentation stream (RAP) consisting of 20 items (distracters and targets) was presented binaurally via Beyerdynamic DT770 Pro circumaural headphones. Each item in the stream lasted for 150 ms followed by an inter-stimulus interval of 10 ms, yielding a presentation rate of 6.25 Hz. T1 was randomly selected from the pool of pure tones (on 50% of the trials) and noise bursts (50% of the trials), and was placed in either the 5th or 7th serial position in the auditory stream. While T1 was presented on every trial, T2 was randomly presented only on 50% of the trials and its pitch and lag from T1 were randomly varied. T2 could appear at Lag1, Lag2, Lag3, or Lag9 following the T1 (corresponding to 160, 320, 480, or 1440 ms of stimulus onset asynchrony between the targets, respectively). When present, in 32 trials T2 was a cello tone, in 32 trials it was a human voice (i.e., sung tone), and in 32 trials it was an organ tone.

At the end of each trial, participants were first asked to identify the first target with a two-alternative forced choice method (pure tone or noise) and then to detect the second target item (e.g., “Was a human voice present or absent following the first target?”). Responses were given by pressing the corresponding keys on the computer keyboard. Participants were instructed to take their time in making their responses.

### 2.4. Musical Sophistication Index

Self-report inventory of the Goldsmiths Musical Sophistication Index (Gold-MSI version 1.0; Müllensiefen et al., 2014) was used to quantify participants' self-reported musical sophistication. The Gold-MSI inventory comprises five sub-scales (active engagement, perceptual abilities, musical training, singing abilities, and emotion) and one general factor (i.e., musical sophistication). It contains 38 items rated on a seven-point scale (and an additional question of the instrument played best). The Gold-MSI inventory is suited for measuring musical engagement and behavior in the general population (i.e., not restricted to the musically trained group).

All scales of the Gold-MSI inventory has been shown to have a good internal consistency (Cronbach's alpha = 0.926 for the general musical sophistication factor, 0.872 for active engagement, 0.873 for perceptual abilities, 0.903 for musical training, 0.870 for singing abilities, 0.791 for emotions; Müllensiefen et al., 2014) a very high re-test reliability (ranging between 0.857 and 0.972; Müllensiefen et al., 2014). The inventory has been demonstrated to have convergent validity with the musical aptitude subscale of the Musical Engagement Questionnaire (MEQ; Werner et al., 2006) and discriminant validity with the less related MEQ subscales like affective reactions (*r* = 0.449, *p* < 0.01 and *r* = 0.182, *p* < 0.05, respectively).

### 2.5. Letter-Number Sequencing Task

Letter-number sequencing (LNS) is a supplemental subtest of the Weschler Adult Intelligence Test (Wechsler, 2008). The LNS is a complex WM span task which involves hearing a sequence of numbers and letters, and then reporting back the numbers in ascending order and the letters in alphabetic order. The task requires not only relying on attention and auditory memory but also manipulating auditory information. Together with the digit span, LNS was found the most highly related psychometric tests to laboratory WM measures (Shelton et al., 2009). Fluid intelligence and cognitive flexibility have also been argued to be involved by this task (Pezzuti and Rossetti, 2017). The individual differences in the temporal costs of selective visual attention has been previously linked to WM capacity and fluid intelligence was associated with higher target accuracy (Colzato et al., 2007; but see Martens and Johnson, 2009 for contrasting evidence) using other tasks (e.g., Operation Span task, Symmetry, and Reading Span tasks as measures of WM capacity, and Raven's Progressive Matrices to assess fluid intelligence), but not with LNS complex span task. It is therefore interesting to explore whether the performance in LNS could be linked to individual or group level differences in the auditory AB. The auditory nature of the task makes it additionally suitable for this study.

### 2.6. Data Analyses

Performance on the T1 identification task was assessed using a repeated-measures analysis of variance (ANOVA) with a between-subjects variable of Group (novice, expert) and the within subject variables of T2 Type (voice, cello, organ) on T1 accuracy data. To explore the AB effects, T2|T1 accuracy (i.e., the accuracy of T2 detection when T1 was correctly reported) data were analyzed using repeated-measures ANOVAs with a between-subjects variable of Group and the within subject variables of T2 Type and Lag. Split-up ANOVAs were performed when interactions were observed. Greenhouse-Geisser and Huynh-Feldt corrections were applied when the sphericity assumption was not met. The significance threshold was set to *p* < 0.05 for all tests. In addition, Bayesian statistical analyses were conducted to determine the evidence proportion in favor of the null (i.e., the absence of an effect, H*0*) and alternative (H*1*) hypotheses. Bayesian statistics were computed using JASP (JASPTeam, 2018; jasp-stats.org). For all the reported Bayesian ANOVAs, the default prior of 0.5 was used for the r-scaled fixed effects as no other information was available to update this prior.

Bayes factors (BF*10*) reflect the likelihood of the data to arise from alternative model (H*1*) in comparison to the null model (H*0*). In some of the analyses with a priori contrasts, the alternative hypothesis was formulated as a directional hypothesis and the Bayes factor reported in these analyses as BF-0. Bayes Inclusion Factor across matched-models (also known as Baws factor; Mathôt, 2017) was used when reporting and interpreting the results obtained from the Bayesian ANOVA with the multi-factorial models design. Bayes Inclusion Factor (BF*Inclusion*) across matched models reflects the evidence for all models that includes a certain effect to equivalent models stripped of that effect. The present interpretation of the Bayes factors for evidence is based on the recommendations by Dienes (2014). Especially, when the BF is comprised between 0 and 0.33, one can confidently conclude in favor of the null model (i.e., no difference). When the BF is between 0.33 and 3 the evidence is inconclusive and no specific statement can be made about which model is supported by the evidence. When BF is 3 or above (to infinity), we have conclusive evidence for a difference. Importantly, the Bayesian approach allows to specify the relative probability that either the alternative or null hypotheses are true. We also consulted Lee and Wagenmakers (2014)'s adjustment of the Jeffreys (1961) original labeling of the evidence categories, which can be found in Supplementary Material.

## 3. Results

### 3.1. T1 Accuracy

The overall performance on T1 identification was high across all three T2 conditions (90, 89, and 88% in voice, cello, and organ conditions, respectively). A repeated measures ANOVA showed a significant main effect of Group, *F*_(1, 34)_ = 12.03, *p* = 0.001, $\eta_{p}^{2}$ = 0.261. *Post-hoc* contrasts revealed that expert cellists were better at correctly identifying T1 than the novices (Mean difference = −0.136, *SE* = 0.039). The main effect of T2 Type, *F*_(2, 68)_ = 0.751, *p* = 0.476, $\eta_{p}^{2}$ = 0.022 and the interaction of T2 Type and Group was not significant, *F*_(2, 68)_ = 1.074, *p* = 0.347, $\eta_{p}^{2}$ = 0.031. As non-significant results cannot be interpreted as a conclusive evidence for the absence of an effect (Dienes, 2014), Bayesian analyses were necessary. As shown in Table 1, Bayesian testing on T1 accuracy scores indicated a moderate evidence supporting that T1 accuracy is 6.17 times more likely to not be affected by T2 Type (BF*Inclusion* = 0.162). This speaks against the differential processing of T1 in the context of voice, cello, and organ detection. Similarly, the null model received moderate support in explaining the data as compared to the interaction of the two factors, BF*Inclusion* = 0.307. Finally, the estimated Bayes factor revealed a strong evidence supporting that the differences in T1 accuracy was about 20 times more likely to occur under the model with participant group than the null model, BF*Inclusion* = 20.365.

**Table 1:** Bayesian analysis of variance across matched models with T2 Type (voice, cello, organ) and Group (novice, cellist) on T1 accuracy scores.

**Effects**	***P* (incl)**	***P* (incl|data)**	**BF incl**
**Analysis of effects**			
T2 type	0.400	0.131	0.162
Group	0.400	0.916	20.365
T2 type × group	0.200	0.038	0.307

*The analysis compares models that contain the effect to equivalent models stripped of the effect. Higher-order interactions are excluded*.

### 3.2. T2|T1 Accuracy

T2|T1 performance analysis (based on trials in which T1 was correctly identified) as a function of lag is the attentional blink effects critical to this study's aim. Results of three-way ANOVAs on T2|T1 performance across all lag conditions are summarized in Table 2. In the following analyses, we focus on the T2|T1 accuracy at Lag 3 (inside the typical AB time window) and at Lag 9 (outside the AB time-window). Lag 3 was selected due to yielding the lowest performance among the lags within the typical AB period (<500 ms). If we were to observe an attentional blink, it would be where the T2 deficit is largest within the typical AB period, and that it would recover outside of this time window.

**Table 2:** Summary of the three-way ANOVA results on T2|T1 accuracy for all lag conditions using Traditional (reported with *p*-values) and Bayesian (reported with Bayes Factor estimations) statistics.

	**Effect**	**Statistics**	***p*-value**	**Bayes factor**	**Evidence category**
Lag 1 vs. Lag 9	T2 type	**F**_(2, 68)_ = 8.01	*p* <0.001	BF = 2035	Extreme evidence for H1
	Group	*F*_(1, 34)_ = 13.37	*p* <0.001	BF = 34.05	Very strong evidence for H1
	T2 type × group	**F**_(2, 68)_ = 6.97	*p* = 0.002	BF = 1,940.62	Extreme evidence for H1
	Lag	*F*_(1, 34)_ = 0.29	*p* = 0.59	BF = 0.153	Moderate evidence for H0
	Lag × group	*F*_(1, 34)_ = 0.35	*p* = 0.56	BF = 0.210	Moderate evidence for H0
	T2 type × lag	*F*_(1.75, 59.44)_ = 1.58^a^	*p* = 0.22	BF = 0.186	Moderate evidence for H0
	Lag × T2 type × group	*F*_(1.75, 59.44)_ = 1.60^a^	*p* = 0.21	BF = 0.305	Moderate evidence for H0
Lag 2 vs. Lag 9	T2 type	*F*_(1.76, 59.70)_ = 9.89^a^	*p* <0.001	BF = 399.39	Extreme evidence for H1
	Group	*F*_(1, 34)_ = 17.59	*p* <0.001	BF = 84.10	Very strong evidence for H1
	T2 type × group	*F*_(1.68, 57.07)_ = 10.35^b^	*p* <0.001	BF = 1,402.02	Extreme evidence for H1
	Lag	*F*_(1, 34)_ = 0.39	*p* = 0.53	BF = 0.160	Moderate evidence for H0
	Lag × group	*F*_(1, 34)_ = 0.39	*p* = 0.53	BF = 0.244	Moderate evidence for H0
	T2 type × lag	*F*_(1.36, 46.37)_ = 0.15^b^	p = 0.78	BF = 0.099	Strong evidence for H0
	Lag × T2 type × group	*F*_(1.36, 46.37)_ = 0.76^b^	*p* = 0.43	BF = 0.225	Moderate evidence for H0
Lag 3 vs. Lag 9	T2 type	*F*_(2, 68)_ = 9.68	*p* <0.001	BF = 1,236.38	Extreme evidence for H1
	Group	*F*_(1, 34)_ = 17.63	*p* <0.001	BF = 128.94	Extreme evidence for H1
	T2 type × group	*F*_(2, 68)_ = 9.66	*p* <0.001	BF = 4,807.10	Extreme evidence for H1
	Lag	*F*_(1, 34)_ = 1.56	*p* = 0.22	BF = 0.284	Moderate evidence for H0
	Lag × group	*F*_(1, 34)_ = 2.49	*p* = 0.12	BF = 0.684	Anecdotal evidence for H0
	T2 type × lag	*F*_(1.69, 57.62)_ = 0.46^a^	*p* = 0.60	BF = 0.110	Moderate evidence for H0
	Lag × T2 type × group	*F*_(1.69, 57.62)_ = 2.22^a^	*p* = 0.12	BF = 0.370	Anecdotal evidence for H0

a *Huynh-Feldt corrected*,

b *Greenhouse-Geisser corrected*.

A three-way ANOVA with T2 Type (voice, cello, organ), Lag (3, 9), and Group (novice, cellist) showed a significant main effect of T2 Type [*F*_(2, 68)_ = 9.68, *p* < 0.001, $\eta_{p}^{2}$ = 0.222], Group [*F*_(1, 34)_ = 17.63, *p* < 0.001, $\eta_{p}^{2}$ = 0.341], and the interaction between T2 Type and Group [*F*_(2, 68)_ = 9.661, *p* < 0.001, $\eta_{p}^{2}$ = 0.221]. *Post-hoc* comparison of T2 Type using Bonferroni procedure revealed that, on the condition that T1 being correctly reported, the voice targets were more accurately reported than the cello targets (*p* = 0.006). T2|T1 accuracy on the voice targets were also higher than the organ targets but this contrast was at the margin of statistical significance (*p* = 0.71). *Post-hoc* comparison of Group revealed that the cellists had significantly higher overall T2|T1 accuracy than the novices (*p* < 0.01). The main effect of Lag (*p* = 0.22, $\eta_{p}^{2}$ = 0.044) and the interaction effects with Lag (Lag × Group, *p* = 0.12, $\eta_{p}^{2}$ = 0.068; T2 Type × Lag, *p* = 0.60, $\eta_{p}^{2}$ = 0.013; T2 Type × Lag × Group, *p* = 0.12, $\eta_{p}^{2}$ = 0.061) did not reach statistical significance. A parallel Bayesian ANOVA showed anecdotal evidence against the Lag-only model (BF*Inclusion* = 0.284). There was inconclusive evidence against the model with the interaction Lag and Group (BF*Inclusion* = 0.637), while the model with the T2 Type × Lag interaction reflected substantial evidence against the interaction, favoring the null hypothesis roughly 9 times more (BF*Inclusion* = 0.110). There was inconclusive evidence against the model explaining the data with the three-way interaction of T2 Type × Lag × Group (BF*Inclusion* = 0.370). The estimated Bayes factor revealed extreme evidence in favor of the models including the T2 Type-only (BF*Inclusion* = 1236.378), Group-only (BF*Inclusion* = 128.94), and the interaction of T2 Type and Group (BF*Inclusion* = 4807.098) than the null model. The model that received the most support was that of the interaction of T2 Type and Group, which was around 4807 times more likely to explain the data than the null model did.

**Figure 2:**
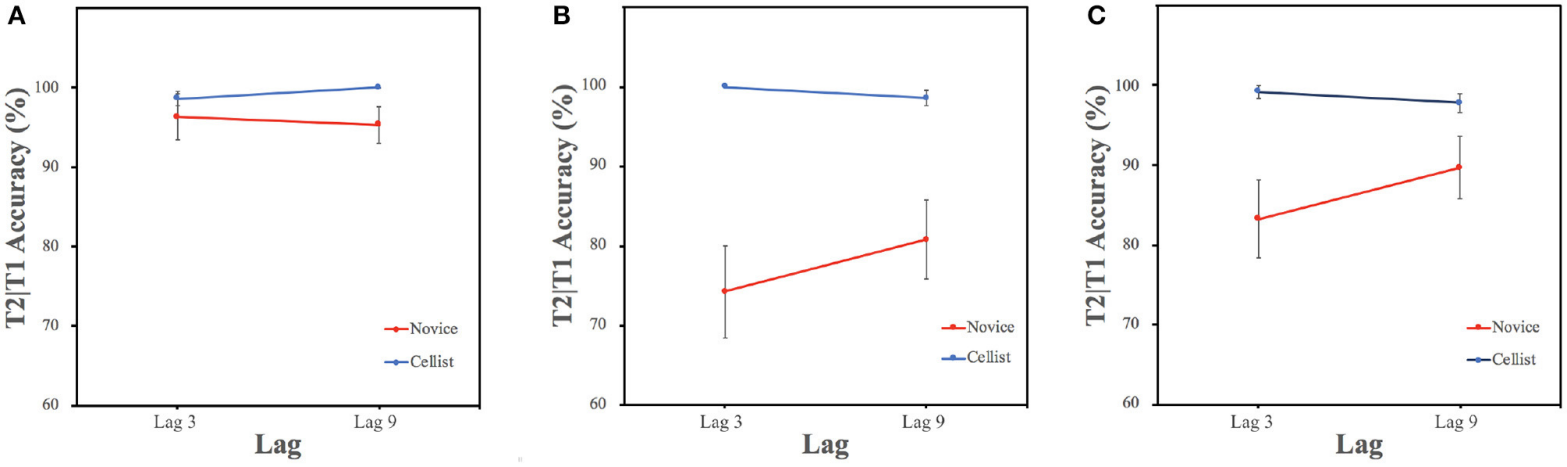
Detection accuracy (%) of the second target (T2) following a correct identification of the first target (T1) as a function of lag across participant groups. Lag 3 and Lag 9 reflect temporal positions within and outside of the attentional blink period, respectively. **(A)** Accuracy ratio (%) when T2 was a human voice, **(B)** accuracy ratio (%) when T2 was a cello tone, and **(C)** accuracy ratio (%) when T2 was an organ tone. Error bars represent standard error of the mean.

Figure 2 illustrates the T2|T1 accuracy percentages of expert cellists and novices under T2 conditions across the two critical Lag conditions (Lag 3 and Lag 9). As visible in the figure, there appears to be an overall benefit of musical expertise across T2 conditions. It also appears that the human voices were less susceptible to the auditory attentional blink regardless of musical expertise. To further explore the two-way interaction of T2 Type and Group, split-up ANOVAs were conducted.

#### 3.2.1. T2 Type Differences on T2|T1 Accuracy

The results of three separate ANOVAs with Lag (3,9) and Group (Novice, Cellist) showed that when T2 was a human voice, the main effect of Group approached the margin of significance [*F*_(1, 34)_ = 3.45, *p* = 0.07, $\eta_{p}^{2}$ = 0.092], while both the main effect of Lag [*F*_(1, 34)_ = 0.01, *p* = 0.91, $\eta_{p}^{2}$ = 0.00] and Lag × Group interaction effect [*F*_(1, 34)_ = 0.38, *p* = 0.54, $\eta_{p}^{2}$ = 0.011] failed to reach statistical significance. A Bayesian ANOVA revealed that the data supported the null model 4.22 times over the model with Lag-only (BF*Inclusion* = 0.237). This is moderate evidence in favor of the absence of the Lag effect. This means that when T2 is a human voice, T2|T1 accuracy rates are more likely to be lag-independent (i.e., no attentional blink effect for human voices). There was inconclusive evidence against the model including the Group as a factor, which suggests a weak change in the odds favoring the null model (BF*Inclusion* = 0.846). Finally, there was an inconclusive evidence against the interaction model of Lag × Group (BF*Inclusion* = 0.404).

When T2 was a cello tone, the two-way ANOVA revealed a significant main effect of Group [*F*_(1, 34)_ = 20.95, *p* < 0.001, $\eta_{p}^{2}$ = 0.381], but the main effect of Lag [*F*_(1, 34)_ = 0.968, *p* = 0.332, $\eta_{p}^{2}$ = 0.028] and the Lag × Group interaction [*F*_(1, 34)_ = 2.30, *p* = 0.189, $\eta_{p}^{2}$ = 0.063] were non-significant. Based on the Bayesian ANOVA model comparisons, the data suggested extreme evidence favoring the Group-only model over the null model (BF*Inclusion* = 365.803), suggesting the variances in the T2|T1 performance accuracy data was 365.803 times more likely to be explained with the main effect of Group over the absence of this effect. The model including Lag as the only factor indicated anecdotal evidence favoring the null model over the Lag-only model (BF*Inclusion* = 0.395). The Lag x Group interaction model decreased the degree of support for the null model compared to the Lag-only model (BF*Inclusion* = 0.687). This means that there was inconclusive evidence favoring the absence of the AB effect in the cello condition and that Group is the most likely candidate in explaining the differences in the T2|T1 performance compared to Lag and the interaction of the two.

When T2 was an organ tone, a two-way ANOVA indicated a significant main effect of Group [*F*_(1, 34)_ = 8.279, *p* = 0.007, $\eta_{p}^{2}$ = 0.196] and a significant Lag × Group interaction [*F*_(1, 34)_ = 4.550, *p* = 0.040, $\eta_{p}^{2}$ = 0.118]. The effect of Lag did not reach statistical significance, *F*_(1, 34)_ = 1.891, *p* = 0.178, $\eta_{p}^{2}$ = 0.053. The estimated Bayes factors indicated that the model received most support against the null model was the Group-only model (BF*Inclusion* = 6.721). The variances in the T2|T1 accuracy data was 6.721 times more likely to be explained with the main effect of Group over the null model. The data suggested anecdotal evidence against the Lag-only model (BF*Inclusion* = 0.529), meaning that there was a slight tendency toward the lag-independence of the T2|T1 performance in the organ condition but the evidence was inconclusive to support this claim further. Finally, there was only inconclusive evidence favoring the interaction model (Lag × Group), BF*Inclusion* = 1.655. The results of two separate Bayes Paired samples *t*-tests with the T2|T1 performances at Lag 3 and Lag 9 showed that while there was a conclusive evidence for no difference for the organ tone detection within and outside the blink periods in the cellist group (BF-0 = 0.117), the evidence remained inconclusive in the novice group (BF-0 = 1.823).

#### 3.2.2. Group Differences on T2|T1 Accuracy

To further explore the group differences, two separate repeated measures ANOVAs with Lag (3,9) and T2 Type (voice, cello, organ) as well as the Bayesian equivalent of the same tests were conducted (see Table 3 for Bayesian results). In the novice group, there was a significant main effect of T2 Type [*F*_(2, 34)_ = 10.178, *p* < 0.001, $\eta_{p}^{2}$ = 0.374] while the main effect of Lag and the interaction effect of T2 Type × Lag were non-significant [*F*_(1, 17)_ = 2.093, *p* = 0.166, $\eta_{p}^{2}$ = 0.110 and *F*_(2, 34)_ = 1.254, *p* = 0.298, $\eta_{p}^{2}$ = 0.069, respectively]. For the same group, a Bayesian ANOVA revealed extreme evidence supporting the model with the T2 Type only (BF*Inclusion* = 4,538.056), which means that the variances in the T2|T1 accuracy among the novices is roughly 4,538 times more likely to be caused by the T2 Type condition than the absence of this effect. The data also suggested anecdotal evidence favoring the null model over the Lag-only model and moderate evidence favoring the null model over the T2 Type × Lag interaction (BF*Inclusion* = 0.472; BF*Inclusion* = 0.266, respectively), meaning that the evidence is inconclusive for lag-dependence while there is moderate evidence against the combined effect of T2 Type and Lag on the T2|T1 accuracy data in this participant group.

**Table 3:** Analysis of effects in novices and in cellists obtained from two separate Bayesian ANOVAs with T2 Type and Lag as variables on T2|T1 performances.

**Effects**	***P* (incl)**	***P* (incl|data)**	**BF Incl**
**Analysis of effects in novices**			
T2 type	0.400	0.921	4538.056
Lag	0.400	0.295	0.472
T2 type × lag	0.200	0.079	0.266
**Analysis of effects in cellists**			
T2 type	0.400	0.979	1213.098
Lag	0.400	0.229	0.306
T2 type × lag	0.200	0.021	0.090

*Both analyses compare models that contain the effect to equivalent models stripped of the effect. Higher-order interactions are excluded*.

In the cellist group, there was a borderline significant T2 Type × Lag interaction effect, *F*_(2, 34)_ = 2.779, *p* = 0.076, $\eta_{p}^{2}$ = 0.141. The main effect of T2 Type [*F*_(2, 34)_ = 0.465, *p* = 0.632, $\eta_{p}^{2}$ = 0.027] and the main effect of Lag [*F*_(1, 17)_ = 0.619, *p* = 0.442, $\eta_{p}^{2}$ = 0.035] were not statistically significant. For the cellists, a Bayesian ANOVA indicated extreme evidence in favor of the T2 Type over the null model (BF*Inclusion* = 1,213.098). This means that the variances in the T2|T1 accuracy among the cellists is ~1,213 times more probable to occur due to T2 type condition than the absence of this effect. There was moderate evidence supporting the null model over the model with Lag as the only factor (BF*Inclusion* = 0.306) and strong evidence favoring the null model over the T2 Type × Lag interaction model (BF*Inclusion* = 0.090). This suggests a moderate support for the lag-independence and a strong support against the combined effect of Lag and T2 Type on the T2|T1 accuracy in this group. To test whether the cellists were better at detecting cello tones than the organ tones a Wilcoxon signed-rank test was performed. The results indicated a borderline statistically significant difference in detecting cello tones and organ tones for the expert cellists (*Z* = 33.00, *p* = 0.042, *r* = −0.614). Median correct detection rates of the cellists were 1.0 for both their own instrument and the organ. Bayes Factor analysis indicated only an anecdotal evidence for this difference, BF*10* = 1.403.

Percentages of T2 hit and false alarm rates for T1-present trials (see Table 4) provide further insight into each groups' performance under different T2 conditions. For cellists, the false alarm rates were generally low (between 1 and 3%) across all T2 conditions. For novices, however, the false alarm rates varied across the different conditions. The rates were especially high under the cello (26%) and organ (16%) conditions, while under the voice condition the rates approached those of the cellists' (3% for the novices, 1% for the cellists). Thus, the results of the novices are highly likely to have been affected by response bias under the cello and organ conditions.

**Table 4:** Percentages of T2|T1 false alarms (FA) and hits.

	**% Hit**			**% FA**		
	**Voice**	**Cello**	**Organ**	**Voice**	**Cello**	**Organ**
Cellists	99.28	99.64	97.97	1.09	2.85	1.27
Novices	97.71	78.88	90.09	3.10	25.81	16.33

### 3.3. Correlation Analyses of the Maximal Attentional Blink Size and the Gold-MSI and LNS Scores

The maximal AB for each individual was calculated (in a similar fashion suggested by Colzato et al., 2007) by subtracting the minimum T2|T1 performance for each individual at an inside the blink period (whichever short lag yields the lowest performance) from the T2|T1 accuracy at the outside the blink period (Lag 9). Maximal AB scores from three cases were identified as outliers (*Z*-scores > 3.29) and were then assigned raw-scores one unit smaller so that they remain deviant but to a lesser extent, following the procedure recommended by Tabachnick and Fidell (2013). Bayesian correlation analyses were then conducted between the maximal AB data across T2 conditions and the Gold-MSI subscales as well as the LNS scores. All reported correlations are measured with Kendall's tau correlation coefficient. Results of these correlation analyses separately for each participant group are summarized in Table 5 together with the mean values and standard deviations.

**Table 5:** Mean scores of the Goldsmith Musical Sophistication inventory (Gold-MSI) and the Letter-Number Sequencing Task (LNS) and Kendall's tau correlation coefficient between the individual's maximal attentional blink size and the Gold-MSI and LNS scores.

	**Novices**				**Cellists**			
	**Mean (SD)**	**Maximal AB voice**	**Maximal AB cello**	**Maximal AB organ**	**Mean (SD)**	**Maximal AB voice**	**Maximal AB cello**	**Maximal AB organ**
Musical sophistication	55.67 (12.23)	0.13	0.06	0.18	108.5 (6.39)	0.40	0.06	−0.39^*^
Active engagement	27.83 (9.13)	0.21	−0.27	−0.06	40.67 (7.02)	0.33	0.30	−0.26
Perceptual abilities	39.11 (7.75)	0.24	0.08	0.04	58.56 (3.67)	0.09	−0.13	−0.48^**^
Musical training	10.78 (3.57)	0.10	0.12	0.16	43.94 (2.04)	0.42	0.22	−0.08
Singing abilities	23.89 (8.80)	−0.04	0.13	0.23	41.17 (3.28)	0.34	−0.04	−0.10
Emotions	31.28 (7.30)	0.15	−0.02	−0.04	36.00 (3.32)	−0.33^*^	−0.06	−0.18
LNS score	9.28 (2.78)	−0.15	−0.16	−0.20	10.22 (2.02)	−0.06	−0.37^*^	−0.24

*For all tests alternative hypothesis specifies that the correlation is negative. Maximal AB = T2|T1Lag9 −T2|T1min*.

* *BF > 3*,

** *BF > 10. Bold values represent conclusive evidence for a negative correlation*.

#### 3.3.1. The Maximal AB and Gold-MSI Correlations

##### 3.3.1.1. The maximal AB and musical sophistication factor

**Figure 3:**
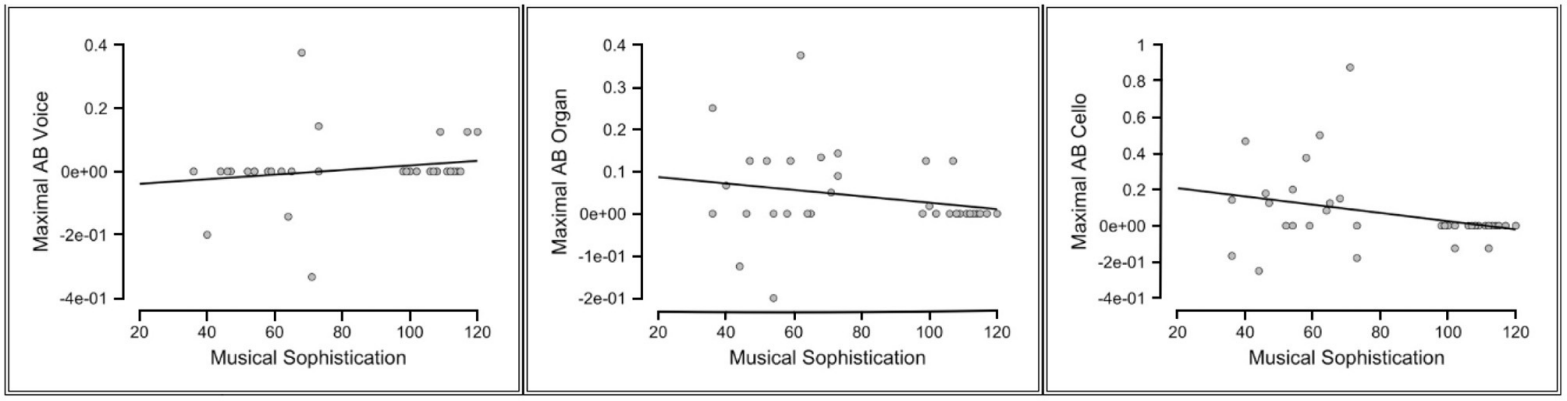
Scatterplots of the relationships between the musical sophistication scores and the maximal attentional blink (AB) in all T2 conditions (Voice, Organ, Cello).

As the maximal AB reflects the largest temporal cost of selective attention for each individual (i.e., the bigger the value for the maximal AB, the larger the temporal cost), assuming that more musically sophisticated individuals would have suffered from the temporal costs to a lesser extent, negative correlations between the maximal AB and the musical sophistication score were expected. For all participants, negative correlations were supported between these two scores only in the cello condition, indicating moderate evidence (Kendall's tau = −0.266, BF-0 = 5.296). The negative correlation between the musical sophistication scores and the maximal AB in the organ condition only had an anecdotal support (Kendall's tau = −0.227, BF-0 = 2.65). The correlation analysis between the musical sophistication and the maximal AB in the voice condition, however, reflected a Bayes factor equaled 0.264 for a one-sided test where H*1* is specified as a negative correlation. This reflects a strong evidence for the null hypothesis. Thus, musical sophistical levels ceased to be relevant in predicting the magnitude of the T2 deficit when T2 was a voice. As illustrated in Figure 3, individuals with relatively higher musical sophistication scores generally showed smaller AB effect in the cello condition, while the maximal AB in the voice condition seems to have no connection with the individual's musical sophistication scores.

##### 3.3.1.2. The maximal AB and the other Gold-MSI factors

The correlations between the maximal AB and the five other factors of the Gold-MSI were also explored using the Bayesian correlation matrix. There was strong evidence indicating a negative correlation between the maximal AB in the cello condition and active engagement factor (Kendall's tau = −0.311; BF-0 = 13.483), as well as moderate evidence for the negative correlations of the maximal AB for cello condition with the factors of perceptual abilities (Kendall's tau = −0.286; BF-0 = 7.878) and musical training (Kendall's tau = −0.274; BF-0 = 6.237). Similarly in the organ condition, moderate evidence supporting the negative correlations between maximal AB and active engagement (Kendall's tau = −0.252; BF-0 = 4.047) and perceptual abilities factors (Kendall's tau = −0.287; BF-0 = 8.145) was found. The strength of all the reported correlations were, however, weak. No other correlations reached a moderate support (BF-0 > 3) under a one-tailed test.

#### 3.3.2. The Maximal AB and LNS Correlations

Bayesian correlation matrix indicated that the correlations between the LNS scores and maximal AB for the T2 Type conditions under the one-tailed test were inconclusive (BF-0 <3). Although when examining the two participant groups separately, we observed that only the data obtained from cellists in the cello condition showed negative correlations between maximal AB and LNS (Kendall's tau = −0.372; BF-0 = 5.192). However, the strength of this negative correlation was weak.

## 4. Discussion

The main purpose of the present study was to determine whether the temporal cost of selective allocation of auditory attention could be lowered or even be eliminated via manipulating the target objects relating to an expert's domain of expertise. Two types of expertise were considered to be interesting to this aim: perceptual expertise with human voices (supposedly common to almost all of us) and musical expertise. The selection of the expert cellists in particular allowed us to test out not only a potential benefit relating to the trained instrument vs. a benefit for all auditory targets (near transfer of the training), but also to explore whether our common perceptual expertise with human voices could possibly have an extended attentional benefit for tones that are acoustically similar to voices (e.g., cello).

### 4.1. Voices Are Least Likely to Suffer From an Auditory AB Effect

The current study demonstrated that neither the instrumental tones of cello or organ nor human voices indicated evidence for the presence of an attentional blink effect, independent of one's musical expertise. The Bayesian analysis allowed us to compare the likelihood of this null effect. Although the evidence was inconclusive to argue for the lag-independence hypothesis (i.e., no support for a temporal cost in the T2 performance) for the cello and organ tones, the evidence supporting the lag-independence was larger (i.e., moderate evidence) in the human voice condition as compared with the cello and organ conditions, meaning that the human voices (when presented as the second target in the RAP) were the least likely to suffer from a temporal limitation to selective auditory attention. This is in line with the hypothesis that human voices as T2 would be less susceptible to suffer an attentional blink effect than the instrumental tones. There are a number of possible explanations that could be argued as to why human voices were the least likely to suffer from an AB effect. A straightforward explanation is that human voices have higher salience than the other target types and they therefore involuntarily capture attention. Hence they are more easily detected in comparison to other targets (i.e., instrumental tones) in this experiment. Weiss et al. (2016) provides physiological support for the heightened salience of voices compared to familiar instrumental timbres, with an observation of greater pupil dilation (reflecting enhanced arousal and stimulus salience) for vocal than for piano melodies. However, given that the AB task by its nature requires voluntary deployment of attention to the targets, the observed attentional benefit for the selection of human voices cannot be explained with the bottom-up salience alone, but rather with an interaction of the top-down mechanisms and bottom-up influences on the temporal selective attention. The interaction between the top-down and bottom up biases is argued to increase the probabilistic competition in the favor of salient target for the object selection (Shinn-Cunningham, 2008), which in this case could explain a more successful detection of the human voices than the other target types. This explanation is consistent with the findings showing other salient targets, such as one's own name (Shapiro et al., 1997) are more likely to survive the attentional blink effect. From an evolutionary point of view, it could even be argued that voices are more salient than one's own name. There is evidence for a very early development of the processing mechanisms for voices and that even fetuses are capable of discriminate human voices from other sounds (Yovel and Belin, 2013). Indeed, it has been reported that the ability to respond to segmented speech develops as early as 36–40 weeks gestational age, and that fetuses can recognize maternal voice (Kisilevsky et al., 2003), which arguably is the most salient auditory stimuli of all due to its evolutionary importance.

The ease of detecting human voices, despite the temporal limitations of the human attentional system, could also be facilitated by the expertise that all humans share when it comes to the processing of human voices. This is in line with other expertise-related benefits that has been observed when the targets are drawn from any objects of expertise. In the sense that voices carry important information about the identity and emotional state of the speaker, they have been referred to in the literature as “auditory faces” (Belin et al., 2004). Using functional magnetic resonance imaging (fMRI), Belin et al. (2000) showed evidence for regions in the human brain that are strongly selective to human voices. These voice-selective areas in the superior temporal sulcus (STS) have been argued to potentially represent the auditory counterpart of the face-selective areas (FFA; Kanwisher et al., 1997) in the visual cortex. In concert with the well-established finding that there is no attentional blink for human faces, the long exposure and experience with the human voices is likely to make all humans capable of processing human voices with no temporal attentional cost. This explanation has support from the previous studies regarding the role of attention in human voice processing. For instance, Levy et al. (2003) found that human voices elicit an ERP component related to Novelty P3 and P3a, suggesting an attentional capture.

It could also be argued that voices may be processed differently than other auditory objects. Beyond the AB paradigm, the overall accuracy rates (across all lag conditions) in the present study was the highest in the human voice condition, which may reflect a processing advantage for voices. In the literature, there are behavioral indications of a processing advantage for voices demonstrated by faster reaction times and lower duration thresholds (e.g., Agus et al., 2010; Agus et al., 2012) in detection and categorization of voices in comparison to the musical instrument tones, with an exception being that this advantage was only observed when RMS-level normalization was used as shown by Bigand et al. (2011). The data in the present study, despite the use of the peak normalization method, indicated a voice superiority in comparison to cello and organ tones reflected in the T2|T1 performance.

The lack of AB for voices may appear inconsistent with some of the findings in the literature using stimuli that also belong to the human voice category, such as spoken letters, spoken digits, and syllables. In the present study, the task required making a type distinction (i.e., reporting hearing a voice), while in the most other studies a token distinction (i.e., reporting what the voice said) was required. Furthermore, in difference from the present study, the distracters in those studies were also typically belonging to the human voice category (e.g., targets were spoken letters, distracters were spoken digits in Martens et al., 2015, and both targets and distracters were spoken syllables in Duncan et al., 1997 and in Tremblay et al., 2005), while in the present study distracters were environmental sounds. By inducing a pop-out effect for human voices presented among environmental sounds, the present study might have a lack of interference between the targets.

### 4.2. The Benefits of Musical Expertise in the Auditory AB Task

In the present study, expertise in music has shown clear benefits in both T1 and T2|T1 performance within the AB task. The current findings supported the lag-independence hypothesis for the expert cellists, as the data showed conclusive evidence for no difference between T2|T1 performances at the inside and outside of the blink periods. Importantly, T2 Type did not modulate the absence of the AB within this group, which speaks in favor of a more generalized auditory attention benefit for the expert musicians in the current auditory AB paradigm.

In all conditions, as expected, the expert cellists had overall higher accuracy rates than the novices. These benefits (reflected by the evidence for group level differences in T2|T1 performance) were extreme when T2 was the cello and moderate when T2 was an organ tone but importantly, this advantage of the expert musicians disappeared when T2 was a human voice. There was a similar trend in the maximal AB scores as well, where the largest difference in the maximal AB scores between the cellists and novices was observed in the cello condition. These data suggested that expert cellists were more likely to have the T2|T1 benefits for their own instrument's tone and some benefit for other instrumental tones in comparison to the novices. This might have happened due to several reasons:

Being more experienced in regards to their principal instrument may have lead to an enhancement of the processing of the tones of that instrument. However, the direct comparison of the differences in cello and organ tone detection rates in cellists only had anecdotal support. To put in another way, although the cellist showed the largest attentional benefits for their own instrument in comparison to the novices, the cellists were almost equally good at detecting organ tones and cello tones. Better allocation of auditory attention for cellists compared to the novices under the cello condition reflects that the cellist did not suffer from the processing costs for the cello as T2 as much as the novices did. Importantly, the highest false alarm rates were observed under the cello condition and the performance of the novices under the cello condition was most likely as a result of guessing. Furthermore, the cellists' auditory working memory span (measured by LNS) was negatively correlated with the processing costs for cello as T2, thus, it is possible that working memory could have a special role in temporal selective attending of the experts for their trained instrument. Motivational and emotional salience of the trained instrument and unintended priming effect (as the cellists knew they volunteered to a study where there was a need for cellist volunteers) may also have an impact on the cellists' performance under this condition.

Although the expert cellists' performance in this study was at the peak level for the detection of human voices just as the instrumental tones, the reason behind this particular finding was most likely beyond their musical expertise, but a rather generalized advantage for all humans, a perceptual expertise for the processing of human voices, since an equally good performance was observed in the novices. This was further supported by the correlation results between the T2 processing costs and the musical sophistication scores in the voice condition.

### 4.3. Anecdotal Evidence Against the AB Effect for Organ Tones

The finding of no AB (although only anecdotally supported) for organ tones in this study was somewhat unexpected. The lag-independence of organ tones was only conclusive in the cellists group, which may reflect a general musicianship advantage. What remains unclear is the reason why the absence of AB for organ tones was inconclusive for the novice participants. Although the T2|T1 performances of the novices were lower at Lag 3 (inside the AB time-window) than at Lag 9 (outside the AB time-window) in organ condition, the slope of this function was not steep enough to indicate an AB effect (in similarity to the cello condition). This could mean that either T2 performance decrement at Lag 3 was too small or that the recovery of this impairment at Lag 9 was not large enough to be captured efficiently by this design. It is possible that the results could be influenced by the parameters used in this study. By altering these parameters, such as presentation rate, stimulus duration, T1 and distracter type, and task difficulty, perhaps it would be possible to obtain conclusive evidence for the temporal costs of attention for instrumental timbres in novices. Alternatively, increasing the power of the study by testing more participants could also help achieving conclusive evidence.

### 4.4. Limitations and Future Directions

The stimulus presentation rate in the present study (6.25 Hz) could be an important factor in explaining our findings. This rate could be too slow to produce a reliable auditory AB effect, as the presentation rate can have dramatic effects on the auditory AB magnitude (Arnell and Jolicoeur, 1999; Shen and Alain, 2010). This may have resulted in giving participants enough time to consolidate the targets into short-term memory. Alternatively, theta entrainment may be an underlying factor for not finding supporting evidence for the presence of an auditory AB effect in none of the conditions tested here. Recently, Shapiro et al. (2017) tested the effects of brain oscillations generated by the rate of the visual presentation stream on the T2 deficit, and found that the visual AB magnitude was the smallest at the theta range (6.26 Hz) in comparison to the alpha (10.3 Hz), beta (16.0 Hz), and gamma (36 Hz) frequency ranges. One future direction for research would be to explore whether an auditory AB effect would occur for voices and instrumental tones as T2 when the stimuli are presented at the rates of the alpha (10.3 Hz) and beta (16.0 Hz) oscillatory frequencies.

Another limitation of our study is that the T2 detection task might have been too easy to capture temporal cost of selective attention, especially in the expert performance. Here the task difficulty was not manipulated to trace individual thresholds, which could also explain the observed ceiling effect in the expert group. It is also possible that the nature of the T1 and T2 task in the design (with a 50–50 chance at obtaining a correct response) made the accuracy data more prone to ceiling effects. Future studies could manipulate the task difficulty based on individual performance and decrease the chance level for correct response to eliminate the potential confound of ceiling effects. Similarly, T1 discrimination task (noise vs. tone among environmental sounds) could also be too easy to produce a reliable auditory AB effect. Several studies investigated the link between T1 task difficulty and the AB magnitude through for example manipulating the effectiveness of the T1 backward masking (e.g., Seiffert and Lollo, 1997; Visser, 2007), response demands of the T1 task (e.g., Jolicoeur, 1999), or T1 perceptual load (e.g., Giesbrecht et al., 2009). Ouimet and Jolicœur (2007) also argued that the data-limited difficulty manipulations (based on low-level perceptual qualities) may be less likely to have a modulatory effect on the AB than the resource-limited T1 manipulations, depending on the impact of the changes in the duration of central processing for this task. So manipulating different factors affecting T1 task difficulty might alter the AB results we have observed in this study.

Finally, as the expert musician population consisted exclusively of cellists, it is difficult to generalize the conclusions to other expert musician groups. However, thanks to this restriction in the participant group, it was possible to contrast the tones of an expert musician's instrument of expertise vs. other instrumental tones that they have not been trained on.

## 5. Conclusion

Under the parameters used in this study, human voices seem to be less susceptible to the auditory AB effects, independent of musical expertise. However, this general effect does not seem to extend to cello tones despite its perceptual similarity to human voices. If this were to be true, we would have observed no group differences. On the contrary, the largest group difference in T2|T1 performance was observed in the cello condition, which reflected an attentional advantage for the expert cellists when the second target was the tones of their principal instrument more than for the other target conditions. Lastly, experts had an overall benefit in the AB task, reflected in both T1 and T2|T1 performances, the exception being the human voice condition.

## Data Availability Statement

All datasets generated for this study are included in the article/Supplementary Material.

## Ethics Statement

The studies involving human participants were reviewed and approved by the Norwegian Centre for Research Data (NSD) on 13 February 2018 [ref. no: 58784/3]. The study was performed in accordance with the recommendations of the Declaration of Helsinki. The patients/participants provided their written informed consent to participate in this study

## Author Contributions

MA conceived and designed the study. MA and BL performed the statistical analyses. MA wrote the first draft of the manuscript. RG and BL edited the parts of the manuscript. All authors contributed to the manuscript revision, read, and approved the submitted version.

### Conflict of Interest

The authors declare that the research was conducted in the absence of any commercial or financial relationships that could be construed as a potential conflict of interest.
