# Supplementary material for: No Evidence for an Auditory Attentional Blink for Voices Regardless of Musical Expertise
Source: Front Psychol. 2020 Jan 10;10:2935. doi: 10.3389/fpsyg.2019.02935 (PMC6966238; doi:10.3389/fpsyg.2019.02935)
Supplement: Supplementary file 1 [file Data_Sheet_1.PDF]

Supplementary Material

1 SUPPLEMENTARY TABLE

Bayes factor	Evidence category
> 100	Extreme evidence for H1
$30 - 100$	Very strong evidence for H1
$10 - 30$	Strong evidence for H1
$3 - 10$	Moderate evidence for H1
$1 - 3$	Anecdotal evidence for H1
1	No evidence
$1/3 - 1$	Anecdotal evidence for H0
$1/10 - 1/3$	Moderate evidence for H0
$1/30 - 1/10$	Strong evidence for H0
$1/100 - 1/30$	Very strong evidence for H0
$< 1/100$	Extreme evidence for H0

Supplementary Table 1 Categorical classification scheme for the Bayes factor interpretation suggested by Lee and Wagenmakers (2013); adjusted from Jeffreys (1961).
